# Neurophysiological insights into sequential decision-making: exploring the secretary problem through ERPs and TBR dynamics

**DOI:** 10.1186/s40359-024-01750-5

**Published:** 2024-04-30

**Authors:** Dor Mizrahi, Ilan Laufer, Inon Zuckerman

**Affiliations:** Department of Industrial Engineering and Management, Ariel University, Ariel, Israel

**Keywords:** Sequential decision-making, ERP (event-Related potentials), TBR (Theta to Beta ratio) dynamics, Neurophysiology, Cognitive strategies

## Abstract

Decision-making under uncertainty, a cornerstone of human cognition, is encapsulated by the “secretary problem” in optimal stopping theory. Our study examines this decision-making challenge, where participants are required to sequentially evaluate and make irreversible choices under conditions that simulate cognitive overload. We probed neurophysiological responses by engaging 27 students in a secretary problem simulation while undergoing EEG monitoring, focusing on Event-Related Potentials (ERPs) P200 and P400, and Theta to Beta Ratio (TBR) dynamics.

Results revealed a nuanced pattern: the P200 component’s amplitude declined from the initial to the middle offers, suggesting a diminishing attention span as participants grew accustomed to the task. This attenuation reversed at the final offer, indicating a heightened cognitive processing as the task concluded. In contrast, the P400 component’s amplitude peaked at the middle offer, hinting at increased cognitive evaluation, and tapered off at the final decision. Additionally, TBR dynamics illustrated a fluctuation in attentional control and emotional regulation throughout the decision-making sequence, enhancing our understanding of the cognitive strategies employed.

The research elucidates the dynamic interplay of cognitive processes in high-stakes environments, with neurophysiological markers fluctuating significantly as participants navigated sequential choices. By correlating these fluctuations with decision-making behavior, we provide insights into the evolving strategies from heightened alertness to strategic evaluation. Our findings offer insights that could inform the use of neurophysiological data in the development of decision-making frameworks, potentially contributing to the practical application of cognitive research in real-life contexts.

**Supplementary Information:**

The online version contains supplementary material available at 10.1186/s40359-024-01750-5.

## Introduction

The secretary problem [1, 2], a pivotal element in optimal stopping theory [3–6], serves as a metaphor for high-stakes decisions under uncertainty. It presents a situation where a decision-maker must choose the optimal time to commit, with each decision eliminating future options. This scenario transcends recruitment, paralleling significant life decisions in diverse contexts such as real estate, finance, and personal relationships. Here, the key challenge lies in the trade-off between acquiring adequate information and the urgency of decision-making, with the risk of missed opportunities juxtaposed against the cost of premature commitment.

This optimal stopping problem is emblematic of decision-making under uncertainty, where the decision-maker faces sequential choices without the ability to revert to previous ones. This framework is particularly resonant in an era rich with information yet constrained by human cognitive capacities [7]. Despite the plethora of data available, individuals often resort to heuristic strategies to manage the cognitive and metabolic costs associated with information processing and decision-making [7].

Our study explores the neurophysiological dimensions of decision-making within the context of the secretary problem, addressing a gap in neuroscientific research, particularly outside of visual search contexts [7–9]. We focus on how neurophysiological markers, especially Event-Related Potential (ERP) components P200 and P400 [10–13] and the Theta to Beta Ratio (TBR) dynamics [14, 15], vary in response to sequential alternatives and influence decision-making processes, due to their relevance to focused attention, cognitive evaluation, and stress, respectively.

The P200 component, associated with attention and orientation toward stimuli, emerges around 200 ms after stimulus presentation. Its amplitude serves as an indicator of attentional engagement, which is pivotal for the initial interaction with the choices presented [10, 16, 17]. The P400 component, manifesting roughly 400 ms post-stimulus, is linked to the cognitive effort and evaluation necessary for processing decision options. This component reflects the depth of cognitive processing engaged to assess each option, highlighting its importance in the decision-making process [18]. Additionally, the Theta to Beta Ratio (TBR) is employed as a measure of stress and cognitive load. An elevated TBR suggests heightened stress levels and cognitive demand, providing insights into the psychological strain experienced by participants during the decision-making task [14, 15, 19, 20]. By incorporating these neurophysiological markers, our study aims to elucidate how individuals allocate attention, engage in cognitive evaluation, and manage stress throughout the decision-making process, contributing to a more comprehensive view of the cognitive and emotional dynamics involved.

The backdrop of our research includes theoretical models that allude to the complexities of the secretary problem. The Adaptive Gain Model [6, 21, 22], for example, illustrates how decision-makers adaptively balance sensory input against urgency signals in varying reliability scenarios, reflecting the dynamic nature of decision-making. Similarly, Linear Threshold Models (LTMs) [23, 24] suggest linearly increasing decision thresholds, offering insight into strategy formation in complex decision scenarios. These models, provide a broader context to understand decision-making strategies that range from linear evidence accumulation to adaptive weighting of information.

Additionally, we draw on recent findings by Sin et al. [25], who emphasize the role of subjective valuation in decision-making. Their research on physiological markers like pupil dilation correlates with internal decision-making challenges and individual choices, highlighting the importance of subjective valuation processes. Specifically, our research aims to explore how these neurophysiological markers inform our understanding of decision-making behavior, particularly in the dynamic setting of sequential offers characteristic of the secretary problem. By examining changes in ERPs and TBR in response to these choices, we seek to unveil the cognitive and emotional dynamics underpinning decision-making strategies in high-stakes, dynamically evolving environments.

To elucidate the neurophysiological processes underlying sequential decision-making in the secretary problem, we formulated specific hypotheses concerning the behavior of these key markers: the P200 and P400 components, and the TBR dynamics. For the P400 component, our hypothesis centers on its role in the cognitive evaluation of each offer. We predict a gradual increase in amplitude across the task, with peaks corresponding to moments of deeper cognitive processing and decision complexity. This pattern would reflect the participants’ engagement in more detailed evaluations as they progress through the offers, particularly as they approach the task’s end and the weight of each decision grows.

Regarding TBR dynamics, we hypothesize an initial variation in response to the task’s onset, reflecting the cognitive and emotional response to beginning the decision-making process. As participants proceed, we anticipate fluctuations in TBR levels, which could reflect changes in cognitive load and emotional regulation. Specifically, we expect these fluctuations to mirror the task’s demands at different stages, with potential increases indicating heightened cognitive effort or stress, particularly in the latter stages of decision-making.

In summary, our study seeks to contribute to the broader understanding of decision-making processes by integrating neurophysiological insights with the conceptual backdrop provided by these theoretical models. Our goal is to deepen the comprehension of the secretary problem, encouraging further detailed and practical research in various decision-making environments.

## Methods

### Participants

The study involved 27 fourth-year students (16 females) from a senior engineering program, ranging in age from 20 to 35 (average age of 24.25 with a standard deviation of 2.0673). Participants were selected for their right-handedness and absence of neurological issues to minimize variability in neurophysiological responses. Informed consent was obtained from each participant, and the appropriate institutional review board approved the study protocol.

### Task design

Participants engaged in six blocks of the secretary problem task, each consisting of 20 monetary offers. These offers, represented as sums of money displayed on-screen, required participants to either accept or reject each one using designated keys (‘y’ for ‘yes’ to accept and ‘N’ for ‘no’ to reject). Simulating the role of apartment sellers, participants were aware that they could neither see future offers nor revisit past ones. The primary objective was to select the highest offer in each block to maximize potential earnings, with their actual compensation (ranging from 100 to 150 NIS) reflecting their performance in the task. It is important to note that if a participant did not choose any offer within a block, it was considered as if they had accepted the last offer presented to them. This approach ensures that every block results in a definitive decision, aligning with the task’s parameters. Details of the offers are documented in Table A1 in Appendix A.

This table is adapted from the study conducted by Hsiao and Kemp [26]. However, in the design of Blocks 2, 4, and 6, an experimental modification was applied where the original bid values from Hsiao and Kemp [26] were increased tenfold. This substantial augmentation of bid values was strategically employed to inject a broadened range of economic scenarios, thereby disrupting any potential conditioning to a constrained set of bid amounts. The purpose of this augmentation was to examine participant behavior in the face of significantly varied bid levels, providing insight into how participants’ valuation strategies adjust when presented with a wider spectrum of potential selling prices for an asset they ‘own’, such as an apartment. The application of increased bid values in alternate blocks is poised to yield insights into the influence of monetary scale on strategy development and risk evaluation. It allows for an assessment of whether, and how, decision-making in the secretary problem adjusts in the presence of larger potential gains, which may mimic real-world economic decision scenarios more closely than a constrained value range.

## Experimental procedure

**Fig. 1:**
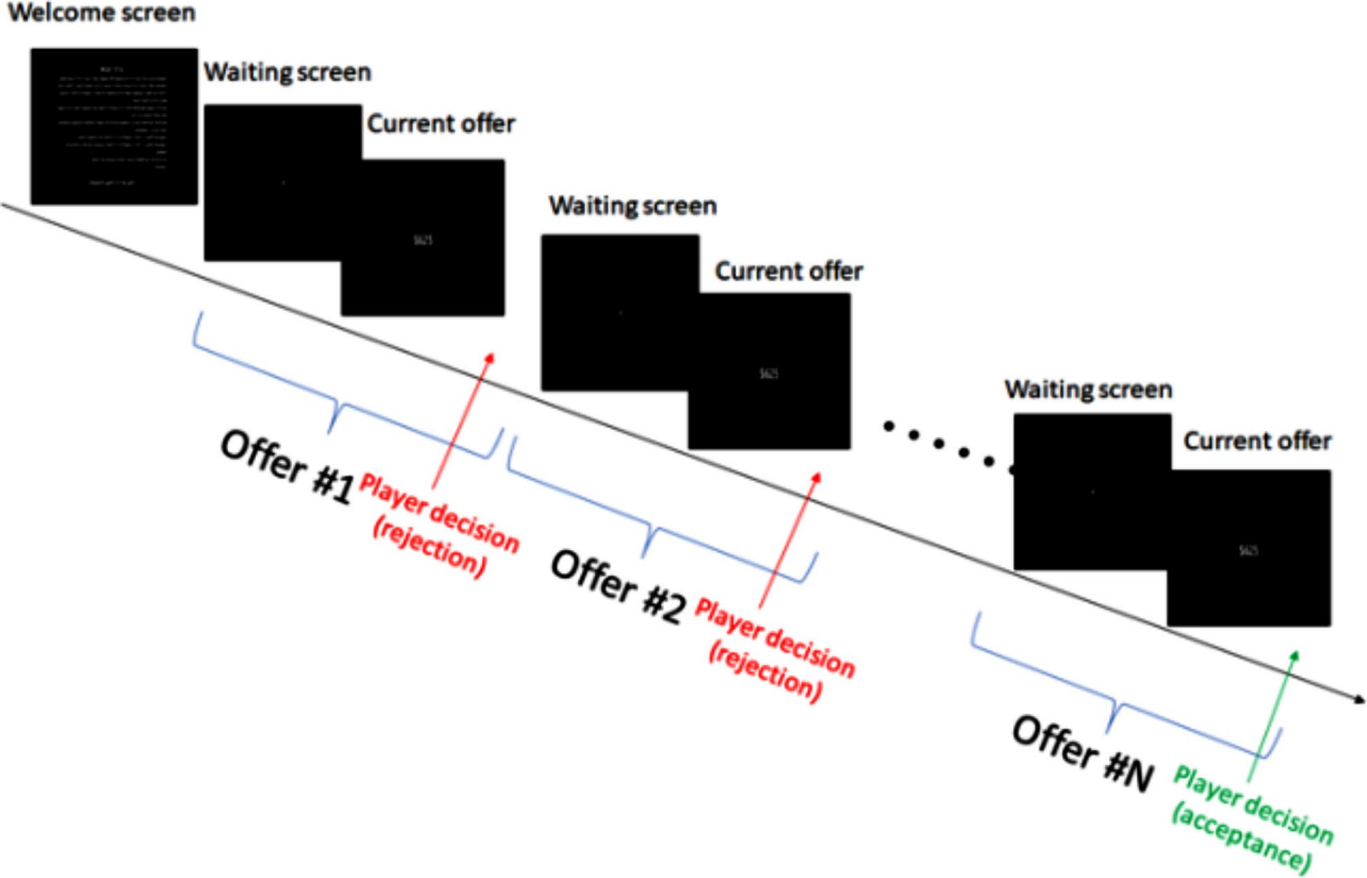
Sequential flow diagram of a single block in the Secretary Problem experiment, depicting offer presentation and participant decision points

The experimental block was structured to reflect the secretary problem, an optimal stopping problem, which allowed us to scrutinize participant decision-making under uncertainty. Each block’s flow, represented in Fig. 1, depicts the sequence of screens and decisions: from the initial welcome screen to the series of offers, punctuated by waiting screens, culminating in the participant’s final decision:

Below, the procedural stages within a single block are outlined as depicted in Fig. 1, encompassing the initial orientation, the intermittent waiting periods, the offer evaluations, and the iterative feedback loop that triggers the cycle to recommence following each decision.

- **Welcome Screen**: This initial display set the context for the upcoming task.
- **Waiting Screen**: Displayed for a randomized duration between 200 and 500 ms, this screen served as a transitional phase, ensuring unpredictability in the timing of the subsequent offer presentation.
- **Offer Screen**: Here, participants were presented with monetary offers, with no time restriction imposed for making decisions. This stage remained active until a decision (accept or reject) was made by the participant.
- **Feedback Loop**: If an offer was rejected and the session had not yet concluded, the procedure reverted to the Waiting Screen, maintaining the cycle of decision-making.

### Incentivization and standardization

The participants’ compensation ranged from 100 to 150 NIS, directly corresponding to their success in selecting the highest offer within the tasks. This tiered incentive system was intended to mirror realistic reward scenarios and to foster a high level of engagement with the task by making the remuneration dependent on the accuracy of their choices. Uniformity across the experiment was maintained by providing all participants with the same sequence of offers.

### Preparation and training sessions

Before beginning data collection, participants underwent a thorough briefing and were equipped with an EEG cap. To ensure they were comfortable with the task and the experimental environment, two training sessions were conducted. These sessions, held without EEG recording, were important for familiarizing participants with the task.

### EEG recording

EEG signals were captured using a 16-channel active EEG amplifier (e.g., USBAMP, by g.tec, Austria) at 512 Hz, adhering to the 10–20 international system. Electrode impedance was maintained below 5 Kohm, monitored using OpenVibe software. Analysis focused on six frontal and prefrontal electrodes (Fp1, F7, Fp2, F8, F3, F7), as these areas correlate with cognitive processing.

Data preprocessing involved a 1–30 Hz FIR bandpass filter and Independent Component Analysis (ICA) to segregate neural activity and artifacts, utilizing EEGLAB’s algorithm for artifact removal, as described by Delorme and Makeig (2004) [27]. In this process, we identified and removed eye movements and blinks, muscle activity from facial and neck muscles, and environmental electrical noise. The latter, often stemming from power lines, was addressed using a notch FIR filter specifically designed to remove such 50 Hz line noise artifacts.

**Fig. 2:**
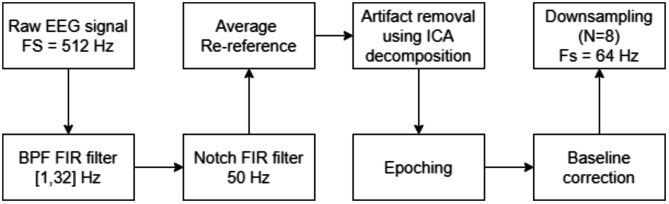
Schematic of EEG preprocessing and analysis pipeline

The following steps in our protocol, illustrated in Fig. 2, involved applying baseline correction 200 ms prior to the onset of the offer screen and implementing average referencing to further enhance the refinement of the EEG data. Downsampling to 64 Hz was strategically reserved for the computation of the TBR, which does not necessitate the high temporal resolution required for the accurate identification of P200 and P400 components’ peak amplitudes. Specifically, the original sampling rate of 512 Hz was preserved for P200 and P400 to accurately capture the temporal dynamics of these faster ERP components. The downsampled data, suitable for assessing the lower-frequency Theta and Beta bands involved in TBR, allowed us to perform this specific analysis with optimal resolution and minimal loss of information.

### ERP analysis and statistical approach

Our study focused on ERPs, particularly the P200 and P400 peaks, to understand the cognitive processes [11] during decision-making in the secretary problem. We analyzed these ERP components for the first, middle, and final offers within each sequence. The first offer refers to the initial one in the sequence, the last offer is the one ultimately chosen by participants, and the middle offer is calculated as the median offer within the range from the first to the last. In cases with an uneven number of offers, the middle offer was identified as the median offer ((total number of offers from first to last + 1) / 2).

For each participant, we identified the maximum amplitude point in each ERP trace within each epoch, corresponding to the initial, middle, and last offers. This approach allowed us to capture the ERP responses specific to each decision point in every block, rather than averaging across all six blocks.

Specifically, we adopted the temporal Regions of Interest (ROIs) as outlined by [11] for the analysis of ERP components, specifically focusing on the P200 and P400. Following their methodology, we identified the maximum peak within these ROIs and then conducted our amplitude calculations within a 60 ms interval surrounding these peaks. This approach closely aligns with the methodology employed by Kornmeier et al. [11], who focused on a ± 20 ms interval surrounding the peaks for their analysis. While Kornmeier et al. [11] averaged the amplitude within these intervals, our study differentiated itself by computing the relative energy across the 60 ms window, allowing for a quantification of the ERP signal’s power within the ROIs.

Kornmeier et al. included two distinct temporal ROIs in their ERP analysis, targeting the P400 between 300 ms and 650 ms, and an earlier P200 effect between 100 ms and 300 ms post-stimulus. We mirrored this approach in selecting our intervals of 170 to 230 ms for the P200 and 370 to 430 ms for the P400 around the respective peaks. With a 512 Hz sampling rate, these intervals of 60 ms surrounding each peak were standardized to correspond to 32 samples, enabling energy calculations surrounding the corresponding peaks.

The decision to utilize frontal and prefrontal electrode placements drew upon the precedent set by visual decision-making studies such as [28], which identified significant ERP activity, including the P400 in frontal regions in the context of a visual Go/ NoGo task. This alignment supports the relevance of our electrode configuration in investigating the cognitive processes mediated by the frontal lobes during decision-making tasks [28].

An essential step in our analysis was establishing a baseline for energy calculations. By assuming a uniform distribution of energy across the entire EEG signal, we calculated a baseline reference value as Relative Energy Baseline (RE_Baseline_) = 32/512 = 0.0625, which is equivalent to 6.25% [13]. The Relative Energy (RE) of the P200 and P400 peaks was calculated by integrating the square of the voltage across the time window corresponding to these ERP components. This provides a measure of the total energy within the segment without baseline correction. The uniform RE_Baseline_ value of 6.25% was then used as a reference to compare the relative energy increase during the P200 and P400 peaks, thereby allowing for the assessment of ERP activations in relation to the baseline EEG activity. To evaluate the impact of different variables on these ERP components, we conducted a two-way repeated-measures ANOVA. This analysis was key to understanding how the offer’s position (initial, middle, end) and the ERP type (P200 or P400) affected the energy patterns observed in each individual epoch.

### Discrete wavelet transform computation for theta and beta band energy assessment

In this study, the TBR was utilized as a critical metric for analyzing cognitive processing across the decision-making sequence [14, 15]. For each participant, the average TBR was computed for each of the six blocks, aggregating all epochs from the first to the selected offer. Consequently, each participant contributed six data points, one from each block, to the analysis.

The Discrete Wavelet Transform (DWT), adhering to established methodologies (referenced in studies [29, 30]), was pivotal in our TBR computation. The DWT’s multiscale representation approach, as detailed in [31], captures various dimensions of the EEG signal. This process incorporated dual digital filters at each stage: a high-pass filter (𝑔(𝑛)) and a low-pass filter (ℎ(𝑛)). Post-filtering, a downsampler with a factor of 2 was employed to adjust the time resolution appropriately. We utilized a 3-level DWT, processing input signals at a 64 Hz sampling rate, effectively isolating coefficients corresponding to the four primary EEG frequency bands. The TBR for each epoch was calculated by measuring the average energy ratio between the Theta and Beta bands (Theta / Beta). Detailed methodology for this calculation can be found in Mizrahi et al. [32, 33].

To further interpret the distribution of these data points, representing averaged TBR for a sequence of offers in each block, a polynomial modeling approach [34] was applied. This method aimed to accurately approximate the TBR values, with each point reflecting the average TBR from the initial to the final accepted offer within a block. Polynomial models of orders one to five were evaluated based on their P-value and R² statistics to determine the most representative model. This analysis allowed us to identify the polynomial order that most accurately mirrored the data trend, offering insightful perspectives on how TBR varied across different stages of offer selection.

## Results

In the next two subsections, we will detail the modulation of P200 and P400 across crucial points in task progression and will elucidate TBR modulation against the position of the accepted offer (last offer) in the task. The ERP components P200 and P400 were specifically assessed at three key stages: the first offer, the median offer, and the final offer received by the participant. These stages correspond to the initial engagement, the development of decision-making strategy, and the final decision point within the secretary problem, respectively. The first offer introduces the participant to the task demands, the median offer represents an intermediary assessment phase, and the final offer captures the participant’s commitment to a decision. In conjunction with ERP data, the TBR was analyzed in relation to the position of the final accepted offer to explore its association with stress levels at the decisive moment of the task. By correlating these neurophysiological markers with task progression, this methodological framework aims to provide insights into the cognitive and emotional aspects that inform decision-making throughout the secretary problem. Data from each of the ERP components (P200 and P400) and TBR values were aggregated across all six experimental blocks and include the data from all 27 participants.

### Dynamics of ERP components across decision stages

**Fig. 3:**
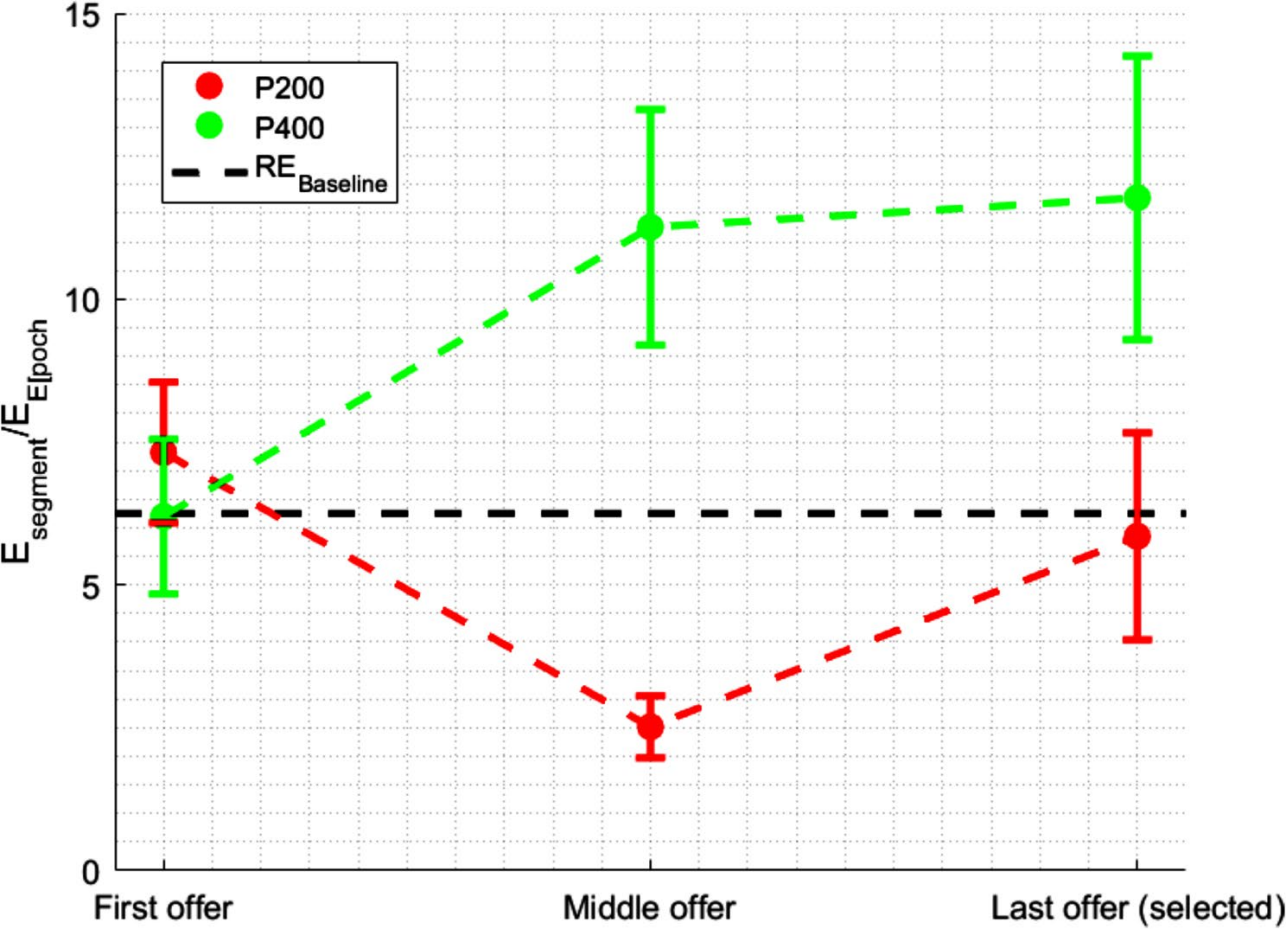
Comparative ERPs across offers, showing P200 and P400 amplitudes at the first, middle, and last selected offers, with standard deviation lines and a reference line indicating the RE Baseline

Figure 3 displays the average relative energies (RE) for the P200 and P400 ERP components at different stages of the secretary problem, with the baseline RE set at 6.25%, as indicated by the dashed line. This baseline signifies a uniform distribution of energy throughout the EEG signal. Figure 3 displays an interaction graph comparing the amplitudes of two Event-Related Potentials (ERPs), P200 and P400, at three pivotal stages of the decision-making task within the secretary problem context. The red trace (P200) begins at a higher amplitude on the first offer, indicating initial cognitive engagement. It then descends to a lower amplitude at the middle offer, suggesting a potential decrease in attentional focus, before ascending again at the last offer selected. In contrast, the amplitude of the P400 component increases from the first to the middle offer, with this upward trend continuing, albeit at a reduced pace, towards the last offer. This pattern suggests a gradual but not uniform intensification of cognitive processing across the task.

Statistical validation, as evidenced by the results of the ANOVA, corroborates the patterns observed in Fig. 3. The analysis revealed significant main effects of offer position on the amplitudes of the ERP components (F(2, 966) = 184.007, *p* < .00001), aligning with the observed shifts in the P200 and P400 traces at different stages of the decision-making task. Specifically, the initial heightened engagement indicated by the P200 amplitude at the first offer, the decreased attentional focus at the middle offer, and the renewed cognitive assessment at the final offer are statistically significant. Similarly, the progression of the P400 component, starting at a lower amplitude and peaking at the middle offer, followed by a decrease at the final offer, is also supported by the statistical analysis.

Furthermore, the ANOVA indicates a significant effect of the type of ERP component on cognitive processing (F(1, 966) = 1831.4, *p* < .00001), reflecting the distinct roles of P200 and P400 in the participants’ cognitive strategies during the task. Notably, a significant interaction effect (F(2, 966) = 750.11, *p* < .00001) highlights the complex interplay between the position of the offer and the specific ERP component. This finding underscores the interdependent influence of these factors on the cognitive processes involved in the secretary problem, suggesting that participants employ shifting cognitive strategies as they progress through the task.

**Fig. 4:**
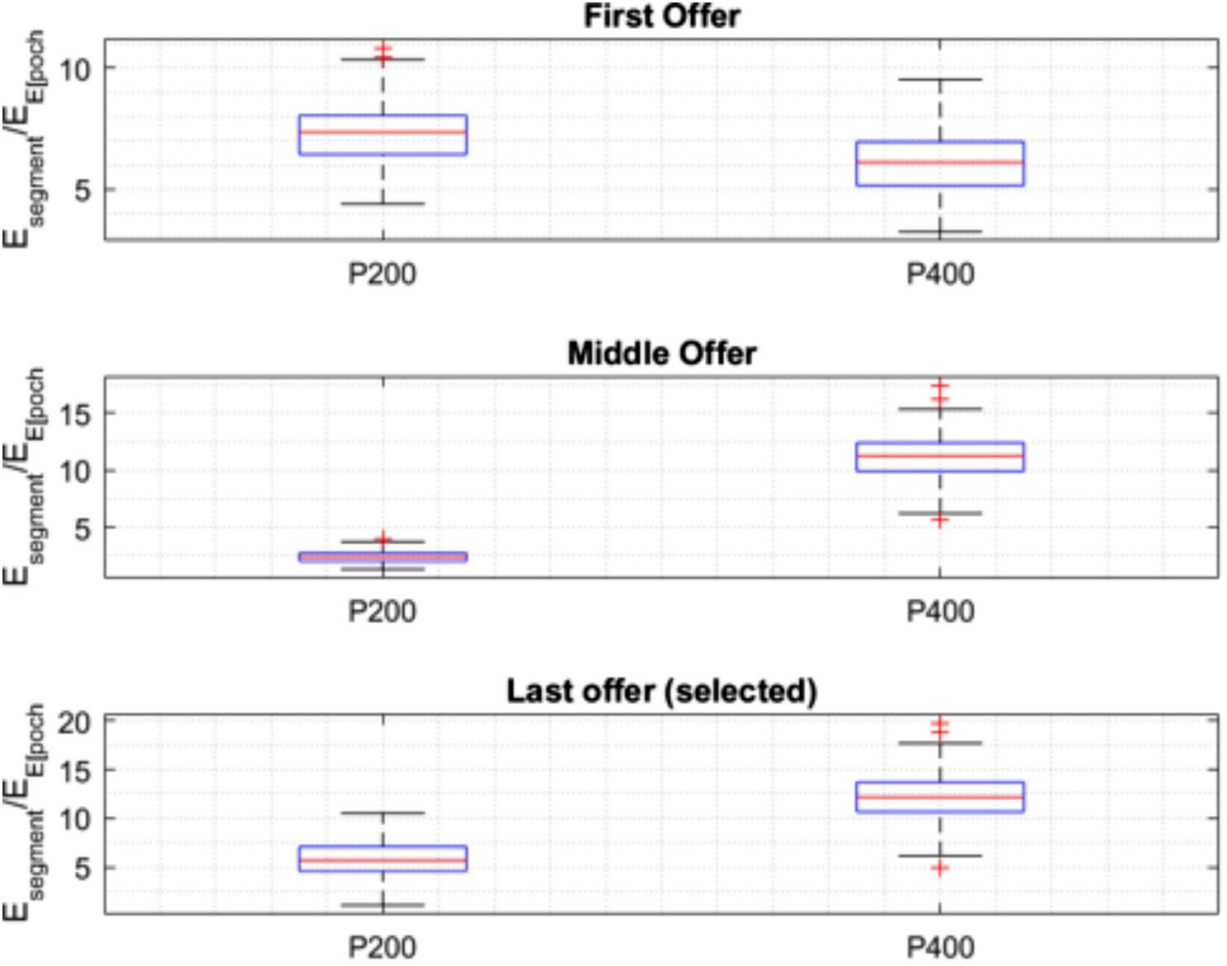
Box plots illustrating median amplitudes and interquartile ranges of P200 and P400 ERPs at the first, middle, and last selected offers in a secretary problem task

Figure 4 offers a visual complement to these findings by presenting the box plots that demonstrate the median and interquartile range for ERP amplitudes of P200 and P400 at three decision-making stages. At the first offer, both P200 and P400 demonstrate the widest range of responses, with P200 showing a notably higher median amplitude. In the context of the secretary problem, the initial offer’s broader range of responses and P200’s higher median amplitude could reflect the exploratory cognitive efforts as participants establish an initial framework for value assessment. The significant difference between P200 and P400 (Estimate: 0.59, 95% CI [1.13, 1.67], *p* < .0001) indicates distinct neural processing as participants encounter the first decision point, which is critical for calibrating their approach to the uncertainty inherent in the task.

At the middle offer stage, the box plot reveals a significant decrease in the median amplitude of P200, alongside a notably narrower spread, evidencing a change in the component’s response from the initial offer. The post hoc test supports this observation, showing a significant difference (Estimate: -9.34, 95% CI [-8.80, -8.26], *p* < .0001). P400’s median amplitude at this stage is observed to be larger compared to P200, with both components exhibiting a reduced variability from their initial responses.

At the final offer stage, data from the final offer stage show an increase in P200’s median amplitude and a broadening of the interquartile range. P400’s median amplitude is also noted to increase slightly, maintaining a substantially higher median than P200. The statistical analysis highlights a significant difference in response at the final offer stage (Estimate: -6.86, 95% CI [-6.32, -5.79], *p* < .0001).

These observations indicate distinct patterns of ERP component responses at key stages of the decision-making task. A decrease in P200’s amplitude and variability is noted at the middle offer stage, followed by an increase at the final offer stage. P400 exhibits an overall increase in median amplitude from the initial to the final stage, with reduced variability across stages.

### TBR dynamics in the secretary problem: indicators of mental stress and decision efficiency

**Fig. 5:**
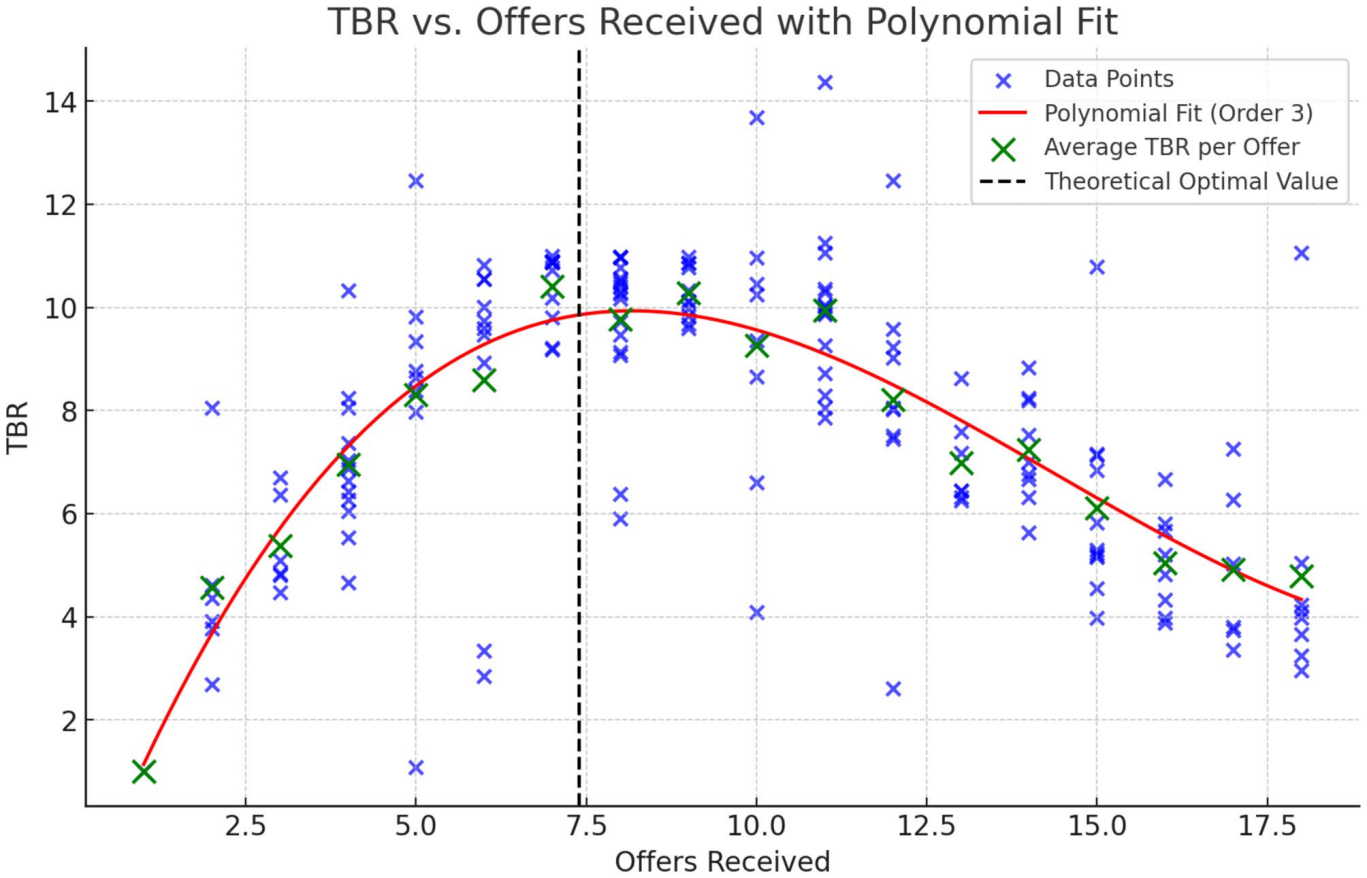
Scatter plot of TBR values by offer sequence number with third-order polynomial fit highlighting decision-making trends in the secretary problem

Figure 5 presents a scatter plot where the individual TBR values, denoted by the small blue ‘x’ marks, are plotted against the sequence number of offers received. A third-order polynomial fit curve, shown in red, captures the overall trend in the decision-making process of the secretary problem. The green ‘X’ marks represent the average TBR value for each offer. The plot reveals an initial phase of lower TBR values, an ascent to peak TBR values slightly after the optimal stopping point—indicated by the dashed vertical line—and a subsequent decline as the offer sequence progresses. This pattern suggests a correlation between the TBR values and the changing levels of executive control, potentially reflecting the different stress levels experienced by participants throughout the phases of the task.

Table 1 provides an analysis of polynomial fits to elucidate the relationship between TBR values and the sequence of offers. It is observed that the third-order polynomial fit, with an R² value of 0.539, offers a substantial improvement in explaining the variance in TBR values compared to the first and second-order fits. While the fourth and fifth-order fits yield a marginally higher R² value of 0.548, the third-order fit is favored due to its balance between simplicity and explanatory power, aligning with the principle of parsimony and avoiding overfitting that higher-order models may entail.

**Table 1:** Analysis of polynomial fit orders and corresponding R² values for TBR data across offer sequences

Polynomial order	*R* ^2^	*p*-value
1	0.039	0.0118
2	0.49	<0.0001
3	0.539	<0.0001
4	0.548	<0.0001
5	0.548	<0.0001

## Discussion

Our study explores the neurophysiological elements of decision-making within the secretary problem, focusing on ERP components P200 and P400, and TBR dynamics. We aim to elucidate cognitive processes and neural markers critical in sequential decision-making under uncertainty.

### Neurophysiological insights into sequential decision-making: integrating ERP and TBR findings

In examining the P200 component during the secretary problem task, we observed a distinctive pattern correlating with the sequence of offers. Initially, there was a marked decrease in P200 amplitude from the first to the middle offers, suggesting a reduction in attentional focus as participants adjusted to the task. However, this trend reversed at the final offer, where a notable increase in amplitude indicated renewed cognitive engagement. This resurgence suggests heightened processing due to the increased significance of the concluding decision. This pattern reveals a more complex cognitive process than just the transition towards a ‘satisficing’ strategy, as posited by Brera and Fu [4]. At first, the elevated P200 amplitude suggests intense scrutiny of early offers. As the task proceeds, the decrease in amplitude at middle offers implies a shift to a ‘satisficing’ approach, where participants accept offers meeting certain acceptable standards rather than only the best. This strategy adapts to task constraints and the need for timely decision-making. However, the increased P200 amplitude at the final offer suggests a strategic reassessment, possibly as participants realize the finality of their choice. This indicates a re-evaluation of their decision against the ‘satisficing’ threshold, demonstrating flexibility in their cognitive approach.

The reduced variability in P200 amplitude at the middle offers, seemingly at odds with the individualistic nature of ‘satisficing,’ suggests a collective adjustment in cognitive engagement due to task structure and constraints. While participants may have personal satisficing thresholds, the task’s design leads to a convergence in cognitive responses. This observation underscores the adaptive nature of decision-making, evolving with the task’s progression and contextual demands.

The P400 component displayed a consistent increase in amplitude throughout the task, reflecting intensifying cognitive effort and strategic evaluation. The significant rise during the middle phase of the task implies an integration of sequential information and adjustment of expectations [12]. This finding is further supported by Hsiao and Kemp’s [26] research on incentive structures in decision-making. Additionally, our interpretation considers the role of internal decision thresholds, akin to ‘internal advice,’ influenced by task incentivization, as illustrated by Liu et al. [5]. Participants’ evolving P400 responses may reflect cognitive adjustments to these internal benchmarks and the task’s incentive structure.

#### TBR dynamics and ERP components: cognitive strategies and emotional regulation

The interplay between TBR dynamics and ERP components sheds light on the cognitive strategies and emotional regulation associated with decision-making in the secretary problem. Lower initial TBR values, which suggest greater executive control and align with findings by Angelidis et al. [15] of enhanced attentional control and reduced stress, set the stage for participants’ strategic information processing. As participants approach the theoretical optimal stopping point, TBR values increase, potentially reflecting heightened cognitive and emotional stress due to the impending necessity of making a critical decision. This rise in stress levels may manifest as an adaptive response, in line with the Adaptive Gain Model proposed by Tickle et al. [6], whereby participants must balance the processing of incoming sensory information with the urgency of making timely decisions. The subsequent reduction in TBR values after this peak could signify a diminution of both cognitive and emotional stress, marking a return to baseline levels of cognitive control.

### Neurophysiology and decision models: conservative choices to digital strategies

Drawing on the insights of Bearden et al. [3] and Babaioff et al. [35], our study’s neurophysiological data suggest a tendency toward conservative, risk-averse strategies in decision-making. This inclination is particularly reflected in the observed patterns of the ERP components and TBR dynamics. For instance, the P200 component’s fluctuating amplitude—initially high, decreasing in the middle, and then increasing at the end of the offer sequence—might indicate a cautious approach as participants evaluate each offer. The initial heightened response suggests careful consideration of early options, while the subsequent reduction could imply a strategic withdrawal of attention from less favorable options, aligning with a risk-averse strategy that avoids premature commitments.

Similarly, the consistent increase in the P400 component’s amplitude throughout the task signals a progressive, in-depth engagement in cognitive processing and strategic evaluation. This pattern could be indicative of participants increasingly scrutinizing each offer’s potential risks and benefits as they move through the sequence, a characteristic of risk-averse behavior where decisions are made more meticulously over time.

The TBR dynamics further complement this interpretation. The rise in TBR values, suggesting heightened cognitive engagement with reduced stress, followed by a decline as participants approach their final decision, aligns with a cautious and measured approach to decision-making. Such a pattern might reflect an increasing awareness of the narrowing options and the consequential nature of the impending final choice, characteristic of a risk-averse decision-making style.

Together, these neurophysiological markers might indicate that participants were exercising a cautious, conservative approach in their decision-making process. This approach, characterized by careful evaluation and minimization of potential loss, has significant implications for designing digital interfaces and decision-making systems. Particularly in online environments where decisions are irreversible, understanding these risk-averse cognitive strategies can inform the development of systems that support users in navigating complex decision-making scenarios effectively.

### Implications, limitations, and future directions

Our study’s findings offer relevant implications for several fields, particularly financial trading and medical diagnosis, where the stakes are high and decisions must be made under uncertainty. In financial trading, understanding the neurophysiological underpinnings of decision-making could lead to the development of tools that help traders better manage stress and cognitive overload, potentially leading to more informed and balanced investment decisions. Similarly, in medical diagnosis, our insights could assist in creating decision support systems that aid physicians in processing complex information under time pressure, enhancing diagnostic accuracy and patient outcomes.

Furthermore, our research holds promise for the development of advanced decision support systems across various domains. By integrating neurophysiological insights into these systems, we can create interfaces that are more attuned to the users’ cognitive and emotional states, potentially leading to more intuitive and effective decision-making tools. Such systems could be particularly beneficial in scenarios where decisions have to be made quickly and with limited information, as they could provide real-time feedback based on the users’ neurophysiological data.

Moreover, our study also highlights the potential for integrating neurophysiological data, such as EEG patterns observed in decision-making scenarios, into artificial intelligence (AI) applications in psychological research and care. This integration could lead to the development of AI-driven decision-support systems that are tailored to respond to the psychological state of users, especially in high-pressure decision-making environments. Our findings suggest that AI, equipped to analyze neurophysiological markers like ERPs and TBR, could significantly enhance decision-making effectiveness in areas such as financial trading and medical diagnosis. This points to a promising intersection of AI and cognitive neuroscience, indicating a future research direction focused on developing AI models that utilize diverse neurophysiological data for practical, real-world applications in psychology and decision-making support.

We recognize the limitations of our study, notably the homogeneity of our participant pool, primarily composed of fourth-year engineering students, and the controlled nature of the experimental setting. These factors constrain the generalizability of our findings. Future research should strive for greater diversity in participant demographics to cover a broader spectrum of cognitive styles and decision-making behaviors. Moreover, incorporating more complex, real-world decision-making scenarios will not only enhance the ecological validity of our findings but also provide deeper insights into the functionality of neurophysiological markers in everyday decision-making processes.

Additionally, our focus on frontal and prefrontal electrodes to capture ERP components P200 and P400 has illuminated the vital roles these brain regions play in decision-making. However, this approach has also limited our exploration of lateralization effects across a broader area of the scalp, potentially overlooking further insights into cognitive strategies employed during sequential decision-making. To address these concerns and broaden the scope of our neurophysiological investigations, we propose an integrated approach for future research.

Future studies could benefit from refining our focus on electrode data analysis to include a comprehensive examination of data from electrodes covering posterior and parietal regions, in addition to the frontal and prefrontal areas. This expanded analysis aims to enhance our understanding of the spatial distribution of cognitive processes in decision-making and allow for a more detailed exploration of lateralization effects across the scalp. Overcoming the spatial resolution limitations of traditional EEG is also critical. Employing high-density EEG systems and integrating findings from fMRI could provide a more nuanced examination of lateralization effects and cognitive process mapping across the brain. This method could offer richer insights into the neural substrates underpinning decision-making tasks. Furthermore, considering the significant impact of physiological and psychological states on decision-making, incorporating alpha band analysis as a complementary metric could prove beneficial. This addition would enable a broader investigation into the interplay between arousal and cognitive processes, enriching our understanding of how these factors interact during decision-making tasks.

In conclusion, our study contributes to the broader understanding of decision-making research, linking neurophysiological data with theoretical and practical applications. Our study aims to contribute to bridging the gap between academic research and real-world applications, hoping that our findings will inspire further exploration and the development of effective support tools and strategies for decision-making in high-stakes environments.

### Electronic supplementary material

Below is the link to the electronic supplementary material.

Supplementary Material 1

## Data Availability

All the experimental data, which includes the players’ electrophysiological recordings and the corresponding resource allocation logs, are stored on the servers of Ariel University. The data can be obtained by request from one of the authors. All the experimental data, which includes the players’ electrophysiological recordings and the corresponding coordination logs, are stored on the servers of Ariel University. The data can be obtained by request from The IRB member, Dr. Chen Hajaj (chenha@ariel.ac.il) or from one of the authors (Dor Mizrahi - dor.mizrahi1@msmail.ariel.ac.il, Ilan Laufer - ilanl@ariel.ac.il, Inon Zuckerman - inonzu@ariel.ac.il).
