# Supplementary material for: Neurophysiological insights into sequential decision-making: exploring the secretary problem through ERPs and TBR dynamics
Source: BMC Psychol. 2024 Apr 30;12:245. doi: 10.1186/s40359-024-01750-5 (PMC11062020; doi:10.1186/s40359-024-01750-5)
Supplement: Supplementary file 1 — Supplementary Material 1 [file 40359_2024_1750_MOESM1_ESM.docx]

**Appendix**

**Appendix A: Bid Distribution in the Secretary Problem**

Table 1: Bid Distribution in the Secretary Problem

| **Offer#** | **Block#1** | **Block#2** | **Block#3** | **Block#4** | **Block#5** | **Block#6** |
| --- | --- | --- | --- | --- | --- | --- |
| **1** | 388 | 7390 | 310 | 4200 | 292 | 4940 |
| **2** | 488 | 8030 | 290 | 6370 | 264 | 2250 |
| **3** | 683 | 2210 | 637 | 7270 | 344 | 2720 |
| **4** | 321 | 7290 | 372 | 5610 | 266 | 9940 |
| **5** | 625 | 1590 | 619 | 6430 | 396 | 6020 |
| **6** | 744 | 1500 | 207 | 6630 | 445 | 9870 |
| **7** | 279 | 2990 | 455 | 5680 | 266 | 5230 |
| **8** | 848 | 8180 | 400 | 6360 | 241 | 6830 |
| **9** | 276 | 5850 | 251 | 4220 | 370 | 14000 |
| **10** | 678 | 8750 | 708 | 3360 | 484 | 15740 |
| **11** | 408 | 1300 | 452 | 4140 | 264 | 14130 |
| **12** | 435 | 7950 | 516 | 4790 | 186 | 1840 |
| **13** | 679 | 4810 | 420 | 3320 | 578 | 10810 |
| **14** | 465 | 200 | 607 | 4940 | 244 | 5580 |
| **15** | 393 | 5250 | 410 | 5460 | 189 | 2730 |
| **16** | 397 | 429 | 324 | 7240 | 565 | 11820 |
| **17** | 588 | 620 | 214 | 4110 | 271 | 3050 |
| **18** | 358 | 4590 | 480 | 2670 | 235 | 6610 |
| **19** | 644 | 7480 | 463 | 3570 | 350 | 7850 |
| **20** | 495 | 3740 | 617 | 3730 | 373 | 890 |

Table A1 presents bid values across six blocks of the secretary problem, with each row corresponding to an individual bid. The table is structured into seven columns: the first column numerically indexes the bids, and the subsequent six columns, labelled Block#1 through Block#6, record the monetary offers made to participants within each block. These columns containing the bid values are the primary data used for our analysis.

Blocks 1, 3, and 5 adhere to the bid values sourced from the study by Hsiao and Kemp [21], however, the bid values used in Blocks 2, 4, and 6 of the study have been increased tenfold from their original figures in the Hsiao and Kemp [21] study. This modification was employed to diversify the range of bids and to prevent the formation of participant expectations based on a limited set of values. The expanded bid range was intended to explore how participants adjust their valuation strategies when faced with a wider array of potential selling prices in a simulated real estate market.
